# Endovascular Infection with *Kingella kingae* Complicated by Septic Arthritis in Immunocompromised Adult Patient

**DOI:** 10.3201/eid2612.191665

**Published:** 2020-12

**Authors:** Mona Mustafa-Hellou, Neta Sagi, Yishai Ofran, Yuval Geffen, Nesrin Ghanem-Zoubi

**Affiliations:** Rambam Health Care Campus, Haifa, Israel (M. Mustafa-Hellou, Y. Ofran, Y. Geffen, N. Ghanem-Zoubi);; Technion Israel Institute of Technology. Haifa (N. Sagi)

**Keywords:** Kingella kingae, bacteria, adult, immunocompromised patient, septic arthritis, endovascular infection, Israel

## Abstract

We report a case of *Kingella kingae* endovascular infection in an immunocompromised elderly patient in Israel who had culture-negative septic arthritis. This case highlights potential sources of metastatic infection other than infective endocarditis, and emphasizes the need for molecular diagnostic methods in detection of pathogens in culture-negative septic arthritis in immunocompromised patients.

*Kingella kingae*, a gram-negative coccobacillus, might be part of the normal flora of the upper respiratory tract. It is a well-recognized causative agent of osteoartricular infections in children <4 years of age (*1*). In adults, it rarely causes infections; the most well-known is infective endocarditis as part of the HACEK (*Haemophilus* species, *Aggregatibacter* species, *Cardiobacterium* hominis, *Eikenella corrodens*, and *Kingella* species) group.

Predisposing factors for *K. kingae* infection are poor oral hygiene, pharyngitis, and mucosal ulcerations (*2*). Few cases of isolated septic arthritis in immunocompetent and immunocompromised adult patients had been described. We report a rare case of endovascular infection caused by *K. kingae* in an immunocompromised adult patient in Israel who had septic arthritis.

## The Study

A 74-year-old woman was admitted to an emergency department because of pain and swelling in her left knee. No fever or chills were reported. She reported no history of a recent invasive procedure, trauma, exposure to animals, or consumption of unpasteurized dairy products. Her medical history indicated that she was a heavy smoker. A high-risk myelodysplastic syndrome had been diagnosed 18 months before her coming to the emergency department. The main manifestations of the myelodysplastic syndrome were anemia, thrombocytopenia, and preserved leukocyte and neutrophils counts. She was given decitabine. She was subsequently given a diagnosis of acute myeloid leukemia and was found to have an isocitrate dehydrogenase 2 gene mutation. Therefore, therapy with enasidenib, an isocitrate dehydrogenase 2 gene blocker, was initiated. She showed a good response for 8 months. Near the time of her admission, the disease progressed, and venetoclax, a B-cell lymphoma 2 inhibitor, was added to her treatment.

**Figure 1:**
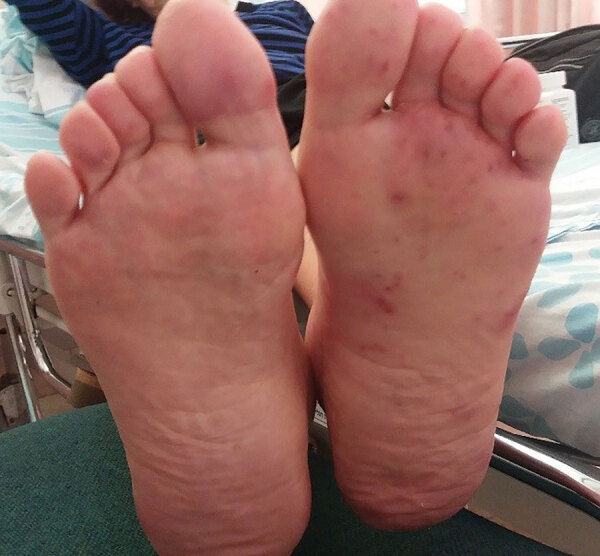
Unilateral, painless, maculopapular, erythematous rash over the sole of the left foot of an immunocompromised patient in Israel with suspected Janeway lesions who had endovascular infection with *Kingella kingae* complicated by septic arthritis. The rash disappeared a few days after initiation of antimicrobial drug treatment.

At admission, the patient was stable hemodynamically and afebrile. Physical examination showed arthritis in the left knee. A unilateral, nontender, erythematous, maculopapular rash was observed on the sole of the left foot (Figure 1). Peripheral pulses were absent distal to left femoral artery, and there were no signs of ischemia of the lower limb. A murmur was heard over the left femoral artery. No heart murmurs were noted on auscultation. White plaques were observed on her tongue and buccal mucosa.

Laboratory tests showed a leukocyte count of 30,000 cells/μL (reference range 4,500 cells/μL–11,000 cells/μL), a hemoglobin level of 8.1 g/dL (reference range 13.8 g/dL–17.2 g/dL), and a platelet count of 22,000/μL (reference range 150,000 cells/μL–400,000 cells/μL). The C-reactive protein level was high (29 mg/dL; reference value <0.3 mg/dL). Blood cultures were drawn before initiating empiric antimicrobial treatment with cefazolin and ciprofloxacin for suspected septic arthritis. An arthrocentesis of the left knee was performed the day after admission and showed a leukocyte count of 83,000 cells/μL with 93% neutrophils. A negative gram stain result was followed by negative synovial fluid and blood cultures.

A PCR was performed for ≈1 mL of synovial fluid. DNA extraction was performed by using the QIAamp DNA Mini Kit (QIAGEN, https://www.qiagen.com) according to the manufacturer’s instructions. Amplification was performed with 2 sets of primers, 1 of ≈500 bp and 1 of ≈1,000 bp, both specific for the 16S rRNA gene. The sets of primers used: set 1, forward: 5′-AGA GTT TGA TCM TGG CTC AG-3′, and reverse: 5′-CCG TCA ATT CMT TTR AGT TT-3′; set 2, forward: 5′-GCA AAC AGG ATT AGA TAC CC-3′, and reverse: 5′-GAC GTC RTC CNC DCC TTC CTC-3′. PCR products were separated by electrophoresis in ethidium bromide–stained 2% agarose gels, and were then sequenced on a 3130 Genetic Analyzer Capillary Electrophoresis DNA Sequencer (Applied Biosystems, https://www.thermofisher.com) and analyzed by using BLAST (https://blast.ncbi.nlm.nih.gov) giving 100% identity to *K. kingae*.

**Figure 2:**
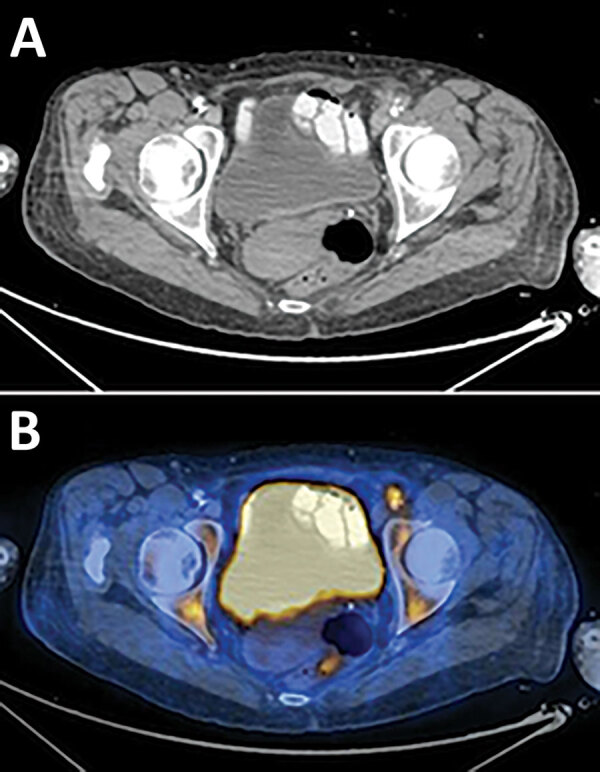
Imaging of an immunocompromised patient who had endovascular infection with *Kingella kingae* complicated by septic arthritis, Israel. A) Computed tomography scan shows a luminal stenosis in the transition zone of the left external iliac artery and common femoral artery along with surrounding turbid fat. B) Fluorodeoxyglucose positron emission tomography–computed tomography scan showing high fluorodeoxyglucose uptake in the plaque causing the stenosis.

Antimicrobial treatment was switched to ceftriaxone. Echocardiography was performed and showed no evidence of vegetation on heart valves. Because of slow clinical improvement and the physical examination findings described, an endovascular focus was suspected. The patient underwent computed tomography angiography of the abdomen, pelvis, and lower limbs, which showed disseminated atherosclerotic plaques in the descending aorta. In addition, a plaque, surrounded by turbid fat, causing a major luminal stenosis was seen on the transition of the left external iliac artery and common femoral artery. Fluorodeoxyglucose positron emission tomography–computed tomography showed high uptake of fluorodeoxyglucose in the plaque in the transition of left external iliac artery and common femoral artery, a finding consistent with endovascular infection and an uptake in the left knee (Figure 2).

The patient was given intravenous ceftriaxone for 6 weeks and showed marked improvement in the C-reactive protein level and leukocyte count. However, 2 weeks later, the patient had a relapse of pain in left knee. Physical examination showed that her left leg was cold, with signs of worsening of arterial insufficiency and minimal swelling of the knee. A repeat fluorodeoxyglucose positron emission tomography–computed tomography showed increased uptake of fluorodeoxyglucose in the left knee, which was suspected for a flair-up of septic arthritis; uptake in the endovascular plaque; and worsening stenosis of the femoral artery. A repeat arthrocentesis trial failed.

Antimicrobial treatment with ceftriaxone was resumed for an additional 6 weeks, which led to improvement of clinical and inflammatory markers. Three months later, the patient showed no further clinical signs of active infection. We also monitored the patient for revascularization caused by worsening arterial insufficiency.

## Conclusions

*K. kingae* usually causes septic arthritis in early childhood (*1*), but has been rarely reported in adults; only 10 such cases were identified in a literature review by using PubMed, Google, and Google Scholar and the words “*Kingella kingae*,” “adult,” and “arthritis.” Previous reports included both male and female patients, of the entire adult age range, and in both immunosuppressed and immunocompetent patients (*3*–*8*). Other reported rare infections with *K. kingae* in adults include peritonitis (*9*), keratitis (*10*,*11*), stomatitis (*12*), urinary tract infection (*13*), and bacteremia (*14*).

The most common infection of *K. kingae* in adults is for persons with infective endocarditis, described in patients with native and prosthetic valves. In some of the cases, there is a clear oral source for the invasive infection. After transient bacteremia, the pathogen seeds heart valves that have damaged endothelium. In our patient, who was a heavy smoker, mucosal ulcers in the mouth caused by candidiasis could have been a port of entry, leading to transient bacteremia and resulting in seeding of the bacterium on the damaged endothelium in an atherosclerotic vascular lesion. The septic arthritis seemed to have been a complication of the infected vascular lesion in the femoral artery, caused either by seeding after bacteremia or after septic emboli to left leg, including the knee and skin of the sole of the left foot.

This case highlights the need for using molecular laboratory techniques to prompt early detection of causative agent in cases of culture-negative septic arthritis, especially in immunocompromised patients with a wide range of potential pathogens, some of which are difficult to identify in the standard laboratory techniques.

In the absence of endocarditis, slow clinical improvement in a patient with septic arthritis caused by *Kingella kingae* should raise suspicion of a deep-seated infections. These infections should include an endovascular source, especially for patients with risk factors for atherosclerosis, such as a history of smoking.
